# Chemical structures and characteristics of animal manures and composts during composting and assessment of maturity indices

**DOI:** 10.1371/journal.pone.0178110

**Published:** 2017-06-12

**Authors:** Jieying Huang, Zixuan Yu, Hongjian Gao, Xiaoming Yan, Jiang Chang, Chengming Wang, Jingwei Hu, Ligan Zhang

**Affiliations:** 1 School of Resources and Environment, Anhui Agricultural University, Hefei, Anhui, People’s Republic of China; 2 Anhui Province Key Lab of Farmland Ecological Conservation and Pollution Prevention, Hefei, Anhui, People’s Republic of China; 3 Institute of Agro-Products Processing Science and Technology, Anhui Academy of Agricultural Sciences, Hefei, Anhui, People’s Republic of China; 4 Hefei National Laboratory for Physical Sciences at Microscale, University of Science and Technology of China, Hefei, Anhui, People’s Republic of China; Old Dominion University, UNITED STATES

## Abstract

Changes in physicochemical characteristics, chemical structures and maturity of swine, cattle and chicken manures and composts during 70-day composting without addition of bulking agents were investigated. Physicochemical characteristics were measured by routine analyses and chemical structures by solid-state ^13^C NMR and FT-IR. Three manures were of distinct properties. Their changes in physicochemical characteristics, chemical structures, and maturity were different not only from each other but also from those with addition of bulking agents during composting. Aromaticity in chicken manure composts decreased at first, and then increased whereas that in cattle and swine manure composts increased. Enhanced ammonia volatilization occurred without addition of bulking agents. NMR structural information indicated that cattle and chicken composts were relatively stable at day 36 and 56, respectively, but swine manure composts were not mature up to day 70. Finally, the days required for three manures to reach the threshold values of different maturity indices were different.

## Introduction

The livestock industry generates huge amounts of manures which contain significant nutrients, organics, heavy metals and pathogens [1–3]. Applications of livestock manures to soil can recycle nutrients, increase soil organic matter, and improve soil physical conditions. On the other hand, hazardous materials such as heavy metals and pathogens potentially lead to environmental contamination. China is one of the largest producers of animal manures in the world, with an annual output of more than 1.9 billion tons [4]. Among all the manures from livestock and poultry in China, swine, cattle, and chicken are the greatest outputs, and thus the main sources of animal wastes [4, 5].

Composting is an environmentally friendly and economical alternative to treating organic wastes and can convert animal manures to organic fertilizers [6, 7]. Whether composts are suitable for land applications depends on their maturity and stability, which can be evaluated by their physicochemical characteristics, and the disappearance of their phytotoxicity assessed by seed germination [8]. Therefore, the knowledge of the physicochemical characteristics, structural changes, and information on seed germination during composting are critical to the understanding of composting processes, evaluation of compost maturity, and facilitation of land applications [9].

Changes in physicochemical characteristics such as C/N, pH, mineral nitrogen, water-soluble organic C, and temperature have been studied during composting. The C/N in the solid phase [10, 11], water extract [12], and water-soluble organic C [2, 10, 12] have been found to decrease as composting proceeded. However, Tiquia and Tam [13] reported increased C/N in poultry litter composts. The pH usually increased with composting [8, 9, 11, 14], but Tiquia et al. [1] found a decline pH trend during 91-day composting of pig litter. The decreased NH_4_^+^-N and increased NO_3_^-^-N often led to low NH_4_^+^-N/NO_3_^-^-N ratios at the end of composting [10, 11, 15]. The composting temperature evolution can be generally divided into three distinct phases, mesophilic, thermophilic, and mesophilic/ maturation stages [7, 11, 13, 14].

Based on physicochemical characteristics, some maturity indices have been proposed to assess compost maturity. Temperature has been regarded as the simplest and most rapid parameter to evaluate the maturity of composts during composting [1, 13]. In addition, C/N, pH, NH_4_^+^-N concentration, and NH_4_^+^-N/NO_3_^-^-N have also been used to assess compost maturity [1, 8, 10, 12]. Most of the chemical characteristics were found to correlate with temperature and with each other [1]. Germination index (GI) was frequently used to estimate phytotoxicity and thus maturity of composts, which is an important criterion for evaluating the suitability of composts for land application. Generally, GI increased during composting [7, 14, 16,17].

A series of spectroscopic techniques such as Fourier transform infrared (FT-IR) and solid-state nuclear magnetic resonance (NMR) have been employed to investigate the chemical structures of manures and composts during composting. Solid-state NMR has been applied to the study of livestock and poultry manures with addition of sawdusts or crop straws [18–20]. Wang et al. [9] compared ^13^C NMR spectra of swine, cow and chicken manures composted with pumice under 55°C, and indicated that O-alkyl-C was the predominant structure in all the three manures, and mineralization of O-alkyl-C dominated the curing stage. Increased alkyl C/O-alkyl C ratios were reported from cattle composts during composting [19]. Kumar et al. [21] evaluated the compost maturity of mixed flower waste vermicompost inoculated with biofertilizer by ^13^C CP/MAS NMR, and found greater aliphatic C than aromatic C. Spaccini and Piccolo [22] indicated that the compost maturity was characterized by a decrease of alkyl components based on ^13^C NMR spectra.

In the literature, organic bulking agents such as straw and sawdust and inorganic bulking agents such as rock phosphate [23] and pumice [9] have been employed to increase porosity and the C/N ratios of composting materials. However, these bulking agents are not used in small- and middle- size composting facilities in China where raw manures are directly composed to reduce cost and labor expenses. Clearly, the transformations of organic matter with and without adding bulking agents are different [9]. The information on the transformations of organic matter during the composting of manures without addition of bulking agents is still limited and the changes of the chemical structures and characteristics are also rarely reported. To the best of our knowledge, only three studies on the transformations of organic matter without any bulking agents during composting have been reported so far, including static-pile composting of pig manure [2] and cattle slurry solid fraction [11], and composting of hog manure [18]. But these studies have rarely focused on the changes of chemical structures during composting.

The objectives of the present study were (1) to study changes in physicochemical characteristics of different manures and composts during composting; (2) to investigate how chemical structures of different manures and composts evolved during composting, and (3) to assess the maturity of composting using maturity indices. To achieve these objectives, FT-IR (details see supporting information) and NMR along with routine physicochemical analyses were employed. Maturity indices based on physicochemical characteristics and chemical structures were compared. Since different manures are of different properties, their changes during composting may differ. Three animal manures, swine, cattle, and chicken manures, with contrasting characteristics, were employed. No bulking agents were added during composting. We hypothesized that the changes in physicochemical characteristics, chemical structures, and maturity varied with the types of manures and were also different from those with addition of bulking agents during composting.

## Materials and methods

### Sample preparation and samplings

Cattle manure was bought from Guihe Animal Husbandry and Fishery Development Co., LTD., swine manure from Wangsheng Breeding Co., LTD., and chicken manure from Daitang Chicken-breeding Farm. The manure composting process was conducted in laboratory. No endangered or protected species were involved. The main chemical characteristics of these starting materials were in Table 1. Three manures were composted separately in a temperature adjustable incubator for 70 days. The details were described in supporting information. The samples of each manure or compost were collected at day 0, 3, 7, 16, 24, 30, 36, 44, 56 and 70 [2,8,17]. A portion of the samples were air dried and ground through a 100-mesh sieve for NMR, FT-IR and elemental analysis, and the remaining portion stored in a refrigerator at– 20°C for other analyses.

**Table 1:** Chemical characteristics of the three manures.

Sample	pH	C(g kg^-1^)	N(g kg^-1^)	C/N	NH_4_^+^-N (g kg^-1^)	NO_3_^-^-N (g kg^-1^)
swine manure	6.63	374.6	46.8	8.0	0.09	4.9
cattle manure	7.86	308.2	28.7	10.7	0.02	0.7
chicken manure	7.73	270.2	53.0	5.1	0.10	2.7

### Chemical analyses

The pH was determined after shaking the manures or composts with distilled water at 1:2.5 (w/v) solid-to-water ratio for 1 hour. Ammonium and nitrate nitrogen were determined by extracting 10 g of samples with 100 ml distilled water (w/v 1:10) by 18-h end-over-end shaking, followed by membrane filtration of the supernatant using 0.45-um cellulose acetate filters. The aqueous extracts were colorimetrically analyzed on a SEAL Auto-analyzer3 [23].

### ^13^C cross-polarization/total sideband suppression (CP/TOSS) and CP/TOSS with dipolar dephasing

The ^13^C NMR spectra were obtained on a Bruker Avance III 400 spectrometer operating at 100 MHz (400-MHz ^1^H frequency). All the experiments were run in a triple-resonance probe head using 4-mm sample rotors. The NMR techniques included ^13^C cross-polarization/total sideband suppression (CP/TOSS) and CP/TOSS with dipolar dephasing (CP/TOSS/DD). The spectra were recorded at a spinning speed of 5 kHz, with a recycle delay of 1.00 s. The 90° pulse lengths were 4 μs for ^1^H, and 4 μs for ^13^C. Sub-spectra for nonprotonated and mobile carbon groups were obtained by combining the ^13^C CP/TOSS sequence with 40-μs dipolar dephasing (CP/TOSS/DD). The number of scans for CP/TOSS and CP/TOSS/DD experiments of all the samples was 4096.

### FT-IR spectroscopy

The chemical structures of manures and composts were also investigated by FT-IR spectroscopy. The details were described in supporting information.

### Germination index

The germination index (GI) was obtained by the method of Zucconi et al. [24] using Chinese cabbage (*Brassica campestris L*.) seeds, which has been shown to be suitable for assessing the phytotoxicity of different kinds of manures and composts. Fresh samples were extracted with distilled water at sample to water ratio 1:10 w/v by shaking for 2 h and then filtered. For the germination experiments, 5 mL of the aqueous extracts were dispensed into 9 mm Petri dishes laid with filter paper. Twenty seeds were placed in each dish and then incubated at 25 ± 2°C in the dark. Experiments were conducted in triplicate and distilled water was used as a control. The seed germination was measured after 3 days. The germination index (GI) was calculated as follows:

$$\text{GI}=\frac{\text{number of seeds germinated in extract}}{\text{number of seeds germinated in control}}\times \frac{\text{mean root length in extract}}{\text{mean root length in control}}\times 100\text{\%}$$

### Statistical analysis

Differences of parameters at different composting stages were assessed with one-way analysis of variance (ANOVA) using SPSS software version 19.0 for Windows. Duncan’s multiple range test was used to determine whether treatments were significantly different from one another. Statistical significance was assigned at the *p*<0.05 level (error probability) for all statistical tests.

## Results

### Physicochemical characteristics and germination index during composting

**Fig 1:**
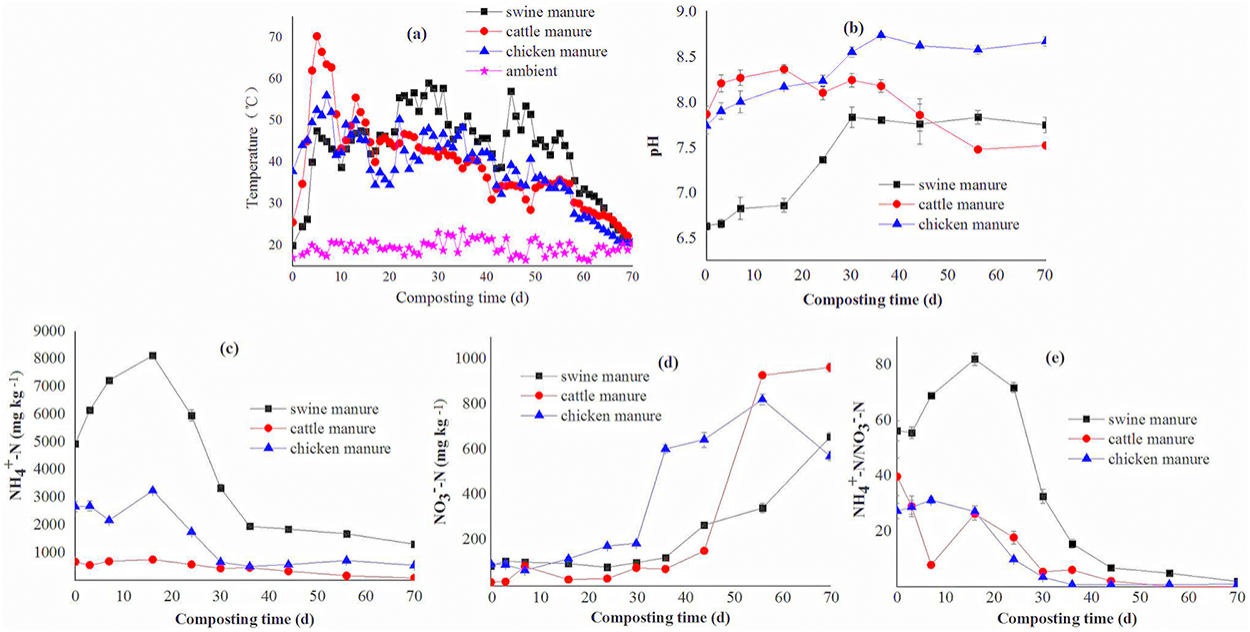
Changes of temperature, pH, ammonium nitrogen [NH_4_^+^-N], nitrate nitrogen [NO_3_^-^-N], and NH_4_^+^-N / NO_3_^-^-N ratio during composting.

Fig 1 shows the changes in temperature, pH, ammonium nitrogen (NH_4_^+^-N), nitrate nitrogen (NO_3_^-^-N), and NH_4_^+^-N/NO_3_^-^-N ratio during composting. The temperature evolutions of all the manure composts followed the same three-stage pattern, the mesophilic, thermophilic, and second mesophilic/ maturation phases (Fig 1(a)). In addition, the results of elemental compositions during composting and FT-IR analysis were described in supporting information (S1 Fig and S1 Table).

The mesophilic phase lasted for 3 days for cattle and chicken manure composts, during which the temperature rose from 25.5°C to 45°C, and 37.7°C to 45.2°C, respectively. But this stage lasted for 5 days for swine manure composts, during which the temperature rose from 20°Cto 47.5°C. In the thermophilic phase, the temperature of cattle manure composts rose to a peak of 70.2°C at day 5 and kept above 50°C for about 10 days between day 4 and day 16. After the peaking, the temperature dropped to 44.7°C by day 16 and then fluctuated between 34 and 46°C from day 16 to day 44. The temperature of swine manure composts increased slowly and reached 55.5°C at day 22, remained at approximately 55°C for about 10 days, reached 57°C at day 45, and then remained above 50°C for about 5 days. The chicken manure composts had a similar temperature change pattern to cattle manure composts, with the thermophilic phase occurring between day 4 and day 14 and the highest temperature only 56°C. The second mesophilic/ maturation phase occurred at day 44 for cattle and chicken manure composts, and at day 56 for swine manure composts.

The pH value of swine and chicken manure composts increased from 6.63 and 7.73 to 7.74 and 8.66 from day 0 to day 70, respectively (Fig 1(b)). Their pH followed a similar trend in their thermophilic phase, sharply increasing from 6.86 to 7.83 for swine manure composts, and from 8.23 to 8.73 for chicken manure composts, respectively, and remaining constant until the end of composting. However, for cattle manure composts, pH increased from7.86 to 8.36 in the thermophilic phase, and then decreased sharply to 7.52 at the end of composting.

The initial NH_4_^+^-N concentration of swine manure was much higher than those of cattle and chicken manures, and increased sharply during the thermophilic phase as a result of the mineralization of organic-N compounds (Fig 1(c)). Then it quickly declined as the composting proceeded. As for chicken manure composts, the NH_4_^+^-N concentration increased obviously in the thermophilic phase, decreased to 662.1 mg/kg at day 30, and then remained almost constant until the end of composting. However, the increase of NH_4_^+^-N concentration of cattle manure composts in the thermophilic phase was slight, but the decrease trend afterwards was almost the same as that of swine manure composts. At the end of composting, only the concentration of NH_4_^+^-N in cattle manure composts decreased below 400 mg/kg, whereas that in swine and chicken manure composts was 1300 and 542.5 mg/kg, respectively. The NH_4_^+^-N in cattle, swine and chicken manure composts decreased by 87.8%, 73.6% and 79.7%, respectively, during the whole composting process. As expected, little NO_3_^-^-N accumulated in three manure composts during the thermophilic phase because nitrifying bacteria were strongly inhibited by temperatures greater than 40°C [11, 25] (Fig 1(d)). The NO_3_^-^-N concentration started to increase rapidly afterwards in all three manure composts. The nitrification occurred during the second mesophilic/ maturation phase, leading to a low NH_4_^+^-N/ NO_3_^-^-N (Fig 1(e)). Also only NH_4_^+^-N/ NO_3_^-^-N ratio of cattle manure composts decreased to 0.1 whereas that of swine and chicken manure composts was 2.0 and 1.0 at the end of composting, respectively. The decreased NH_4_^+^-N content and increased NO_3_^-^-N content in the three manure composts were significantly correlated with the change of pH, and showed a good correlation with temperature change (data not shown).

**Fig 2:**
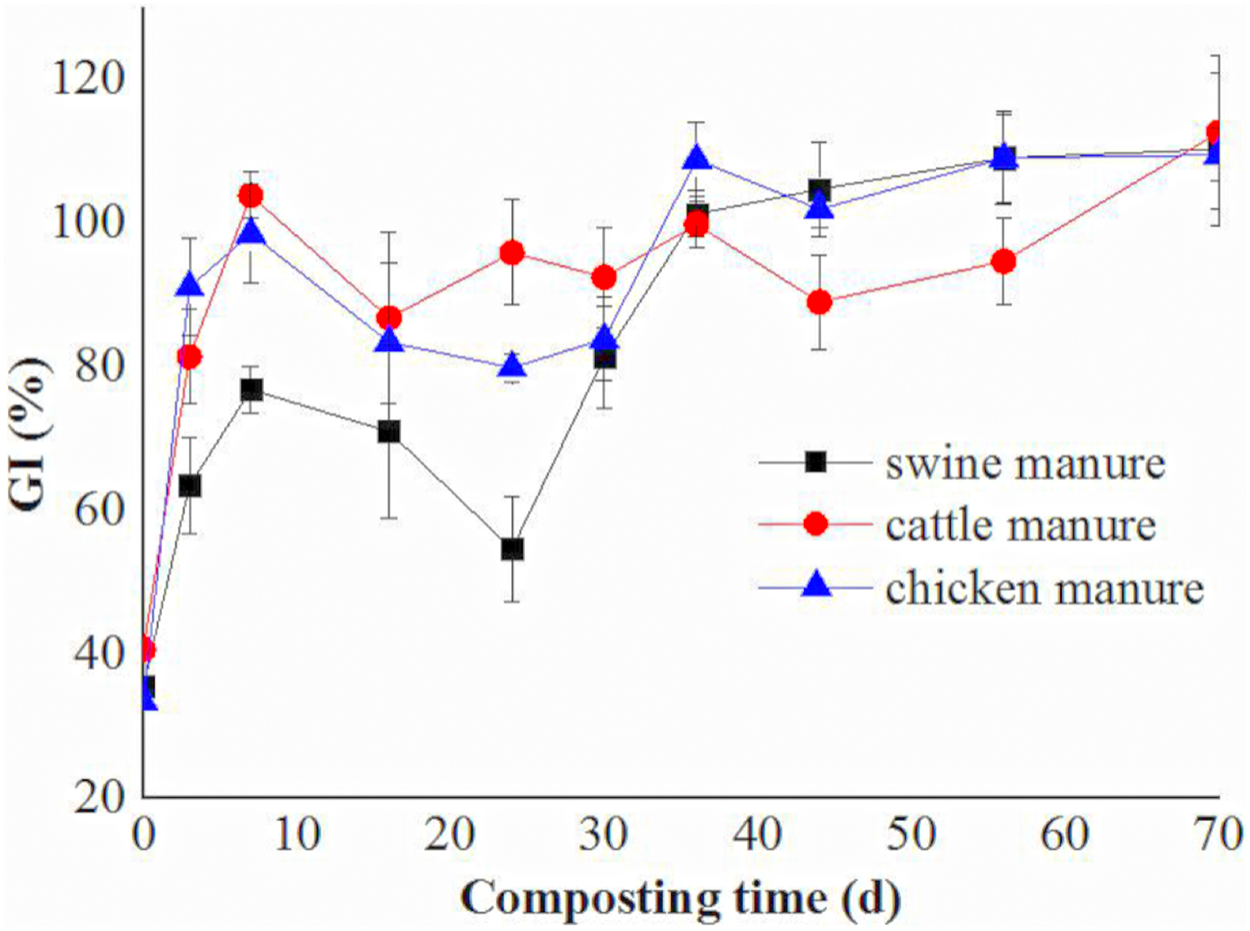
Changes of GI in the swine, cattle and chicken manure composts during composting.

The GI is believed to be a good index of maturity [24]. The change in GI during the composting of the three manure composts is shown in Fig 2. The manures had low GI values which increased rapidly in the first 7 days, and then increased slightly during the late stage of the mesophilic period, due to decomposition of the phytotoxic organic compounds. The GI reached 90% after 24 d for the cattle manure composts but after 36 d for the swine and chicken manure composts. The GI values differed widely among the three manure composts during the thermophilic phase. At the end of the composting, the GI values of three manure composts all reached 100%.

### ^13^C NMR analysis

**Fig 3:**
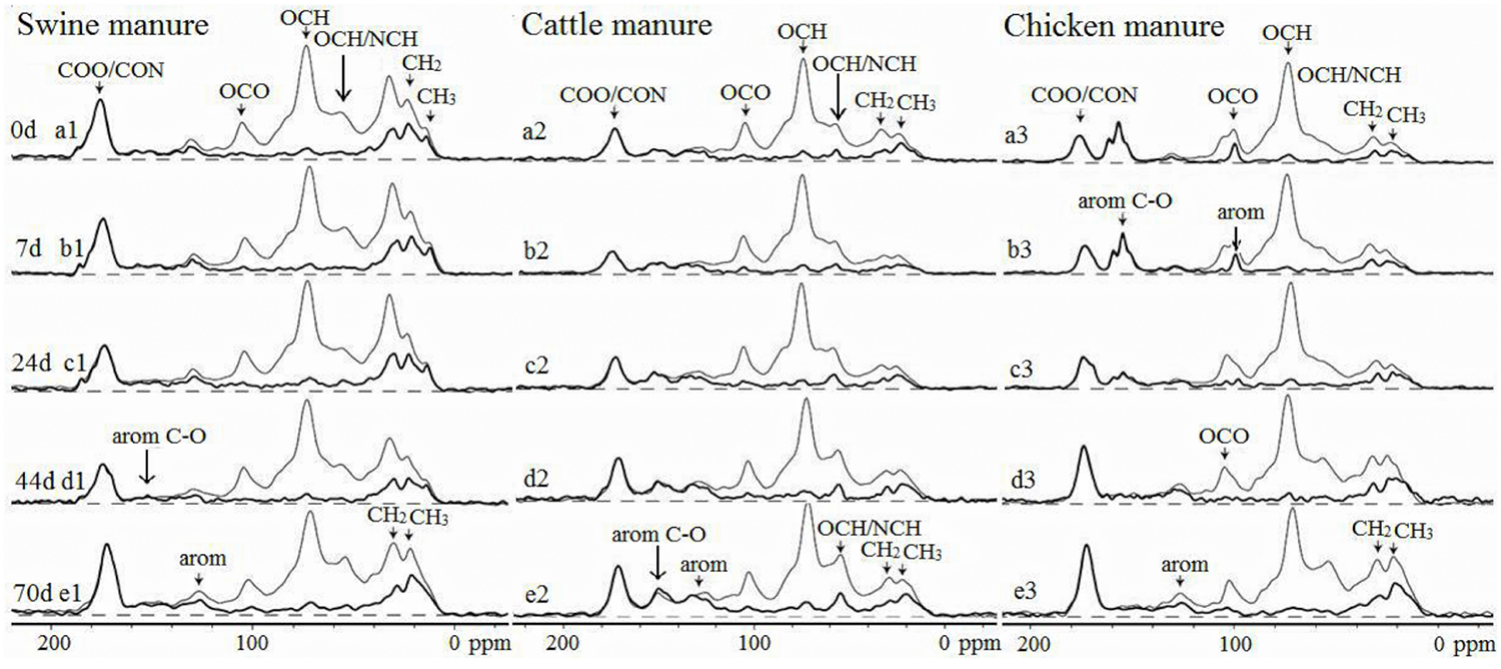
The spectra of ^13^C CP/TOSS [thin lines] and CP/TOSS after dipolar dephasing [thick lines] of swine, cattle and chicken manures and composts [only data of selected days shown].

**Fig 4:**
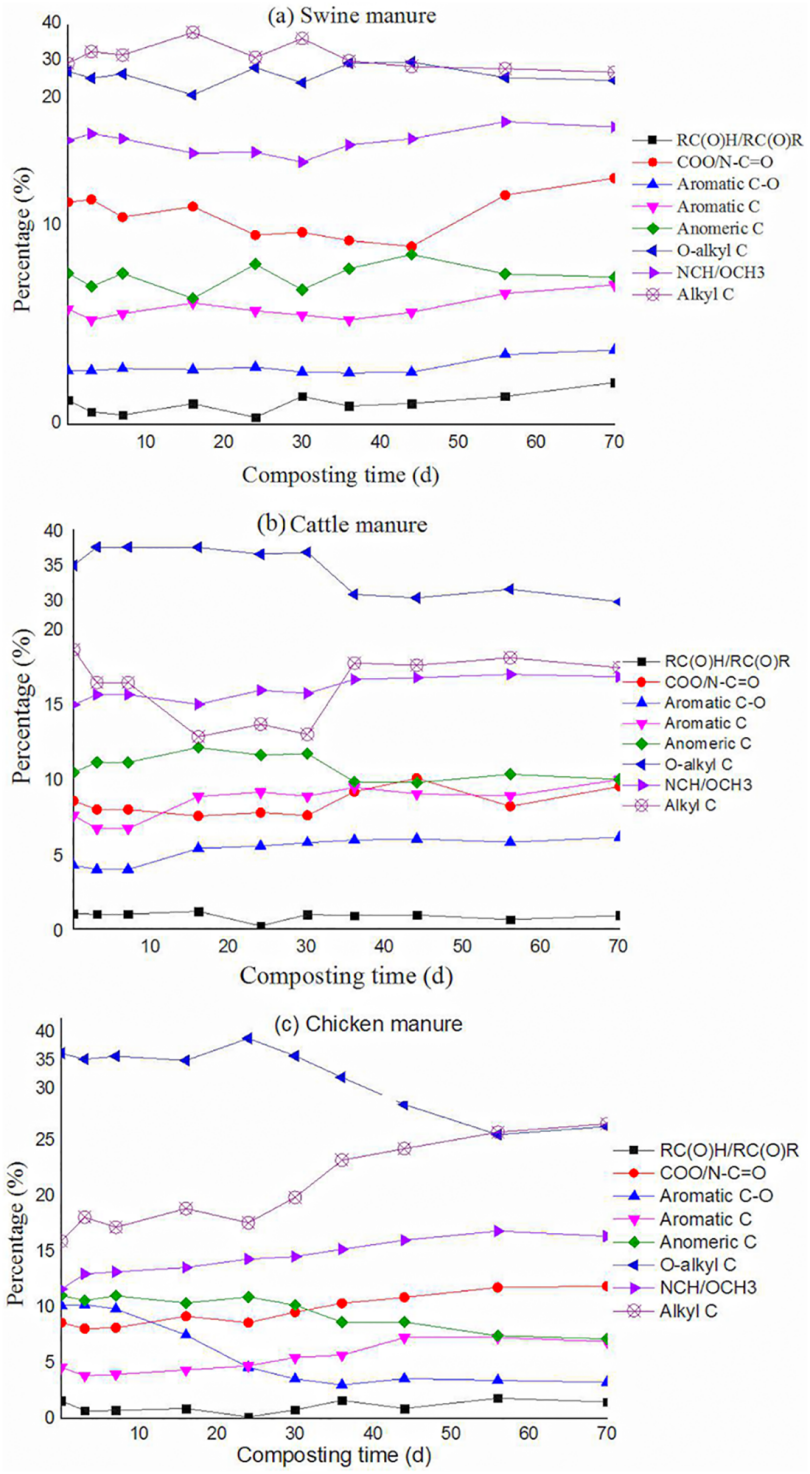
Relative proportions of functional groups [%] in swine, cattle and chicken manure composts obtained by the CP/TOSS and their changes with time.

The spectra of ^13^C CP/TOSS (thin lines) and ^13^C CP/TOSS with dipolar dephasing (thick lines) of manures and composts are shown in Fig 3. Although CP/TOSS is not a quantitative technique, it suffices for comparisons of the structural differences among the manures and their composts during composting. The changes in the relative abundance of different functional groups derived from the spectra in three manures and their composts are shown in Fig 4.

For the ^13^C CP/TOSS spectra of all the three manures (Fig 3a1, 3a2 and 3a3), in the alkyl C (0–44 ppm) region, there were signals around 30 ppm attributed to CH_2_ groups possibly from long-chain polymethylene structures (e.g. fatty acids and waxes) and bands around 22 ppm due to terminal methyl groups from acetyl substituents that may originate from plant hemicellulose [26]. The assignments were confirmed by the corresponding CP/TOSS/DD spectra since these signals were retained due to their fast rotations and thus weak dipolar couplings. In the 44–64 ppm region, the shoulders around 55 ppm could be from both NCH and methoxyl C. However, most of the signals were removed in the CP/TOSS/DD spectra, indicating that the signals in this region were primarily attributed to NCH and there were only small amounts of OCH_3_ functional groups in the manures. The resonances from O-alkyl C (64–93 ppm) were primarily associated with OCH from carbohydrates such as cellulose and hemicellulose and almost no signals from OC_q_ were present, as indicated by CP/TOSS/DD spectra. The bands between 93–113 ppm were assigned to the anomeric C and/or aromatics and the small signals around 113–142 ppm were attributed to aromatic C. The bands in the region of 142–162 ppm were attributed to O-aryl C. Finally, the sharp signals around 172 ppm were assigned to carboxyl groups and/or amide groups.

The ^13^C CP/TOSS spectra of all the three manures are distinct. Compared with the spectrum of swine manure (Fig 3a1), the CP/TOSS spectrum of cattle manure (Fig 3a2) displayed smaller signals from the alkyls (0–44 ppm), and COO/N-C = O (162–188 ppm). In addition, there was a CH_3_ signal at 13.6 ppm in the CP/TOSS spectrum of swine manure but not in that of cattle manure. Compared with the CP/TOSS spectrum of swine manure (Fig 3a1), the spectrum of chicken manure (Fig 3a3) displayed smaller signals from the alkyls (0–44 ppm), O/N-alkyl (44–64 ppm), aromatics (113–142 ppm), and COO/N-C = O (162–188 ppm). Most interestingly, an intense aromatic C-O band around 153 ppm was present in the CP/TOSS spectrum of chicken manure. Moreover, its dipolar dephasing spectrum resolved a nonprotonated band around 102 ppm. These two bands together suggested the presence of polyphenols in the sample. Compared with the CP/TOSS spectrum of cattle manure (Fig 3a2), the spectrum of chicken manure (Fig 3a3) displayed smaller signals from the alkyls (0–44 ppm), OCH_3_/NCH (44–64 ppm), and aromatics (113–142 ppm). Again, the aromatic C−O signal around 153 ppm and the aromatic signal around 102 ppm attributed to polyphenols were considered to be composed mainly of tannins, but they were not present in that of cattle manure [19, 20, 27]. The dipolar dephasing spectrum of cattle manure resolved a nonprotonated band around 56 ppm, suggesting the presence of lignin in the cattle manure.

As the composting proceeded, relative abundances of functional groups changed. O-alkyl C and anomeric C of cattle manure composts increased from 34.8% and 10.4% to 37.4% and 12.1% of total C, respectively, in the thermophilic phase, and then decreased to 29.6% and 9.9% at the end of composting (Fig 4b). In contrast, alkyl C and COO/NC = O decreased from 18.6% and 8.5% to 12.8% and 7.5%, respectively, in the thermophilic phase, and then increased to 17.4% and 9.5%, respectively, at the end of composting. Meanwhile aromatic C and aromatic C-O decreased from 7.5% to 6.7% and 4.2% to 4.0% in the first 7 days, and then increased to 9.9% and 6.1%, respectively, at the end of composting. Compared with cattle manure composts, the changes of functional groups in swine manure composts (Fig 4a) were more complicated. Alkyl C increased from 29.5% to 37.9% in the first 16 days, decreased to 31.2% at day 24, increased afterwards in the thermophilic phase, and then decreased to 27.2% at day 70. Meanwhile O-alkyl C showed an almost reversed changed pattern. The change of COO/NC = O C showed a positive correlation with the temperature change (data not shown). Conversely anomeric C had an opposite change pattern to that of COO/NC = O C. Aromatic C and aromatic C-O fluctuated before day 56 but increased at the end of the composting. The functional group changes in chicken manure composts (Fig 4c) were different from those of cattle (Fig 4b) and swine manure composts (Fig 4a). The alkyl C fluctuated between 16.0% to 18.9% from day 0 to day 24, and then steadily increased up to 26.5% at the end of composting. The O-alkyl C remained around 36.31% from day 0 to day 30, and then steadily decreased to 26.27%. The COO/NC = O and aromatic C decreased from 8.6% to 8.2% and 4.6% to 4.0%, respectively, at day 7, and then increased to 11.9% and 6.9% at day 70. Anomeric C and aromatic C-O successively decreased from 11.1% to 7.2% and 10.2% to 3.3%, respectively, during the 70 days of composting process. In contrast, aromatic C-O increased in the other two manure composts.

## Discussion

### Changes of physicochemical characteristics and germination index of manures and composts during composting

The temperature evolution of three manure composts followed the same pattern as that of other reports [7, 11, 13, 14]. The cattle and chicken manure composts reached the thermophilic temperature faster and had a shorter thermophilic phase than the swine manure composts did. This could be because the swine manure contained the least O-alkyls and anomerics of carbohydrates, and thus its organic matter was more resistant to microbial attacks. Several temperature peaks in swine manure composts could be due to its distinct chemical compositions and microbial communities. Compared with those in the studies with the addition of organic bulking agents [7, 13, 14], the peak temperatures of our three manure composts were much lower. This may be due to the enhanced temperatures from the faster degradation of organic matter due to appropriate C/N ratios adjusted by organic bulking agents. Temperatures greater than 50°C were achieved and sustained over 10 days for cattle and swine manure composts, and sustained over 7 days for chicken manure composts, which ensured maximum reduction of pathogens to meet the maturity and sanitation requirements of composting [14].

pH is an indicator of the state of composting [8]. The pH evolution in cattle manure composts in the present study was similar to that of other studies [7, 9, 11, 14], showing an increasing trend in the thermophilic phase and a decreasing trend in the second mesophilic period. The increase of pH in the thermophilic phase could be attributed to the degradation of acid-type compounds and the increase of ammonia [7]. However, Bustamante et al. [7] observed an initially decreased and then increased pH evolution pattern in exhausted grape marc and poultry manure composts. They ascribed the initially decreased pH to the generation of acid-type organic compounds of low molecular-weight via the decomposition of the most easily degradable organic matter fraction. In addition, Tiquia et al. [1, 13] showed that pH generally decreased during the composting of pig and poultry litter, but they did not provide the mechanism. In the present study, the pH of swine and chicken manure composts sharply increased in the thermophilic phase and remained almost constant until the end of the composting, not consistent with the trend of either our cattle manure or those in the literature [1, 7, 8, 11]. This should be due to the greater amount of ammonia generated in swine and chicken manure composts than that in cattle manure composts.

The NH_4_^+^-N concentration decreased dramatically during composting due to NH_3_ volatilization as the pH was higher than 7. The addition of bulking agents such as superphosphate can adjust pH value, thus reduce NH_3_ emission and improve the nutrient of composts. The NO_3_^-^-N concentration was very low at the beginning of the composting and increased sharply in the second mesophilic/maturation phase. However, the increase was not as pronounced as the decrease of the NH_4_^+^-N. These results are in agreement with the findings of other studies [7, 10, 11, 13]. The NH_4_^+^-N concentration is usually used as a maturity index. Zucconi and Bertoldi [15] proposed a threshold value of 400 mg/kg for a mature city refuse compost. The NH_4_^+^-N/NO_3_^-^-N ratio lower than 0.16 was suggested by Bernal et al. [10] as a maturity index for composts of all origins. In the present study, only the cattle manure compost at the end of the composting had the concentration of NH_4_^+^-N below 400 mg/kg and the NH_4_^+^-N/NO_3_^-^-N ratio below 0.16. This could be ascribed to the lower NH_4_^+^-N and higher NO_3_^-^-N concentrations in cattle manure composts.

The changes of the GI of different manure composts observed in the present study were similar to those of other studies [7, 14, 16, 17], showing an increasing trend with composting time. Zucconi et al. [24] regarded composts with GI values greater than 50% as being absent of phytotoxins. Thus all three manure composts could be safely used in agriculture without any phytotoxic effects. In the thermophilic phase, swine manure composts showed the lowest GI values, correlated with their lowest temperatures and highest ammonium concentrations, which is consistent with the results by Tiquia [16].

### Structural differences of three manure composts during composting process

#### Alkyl-C

In swine manure composts, alkyl-C fluctuated in the theromphilic phase and slightly decreased until the end of composting, which is inconsistent with other studies in the literature [9, 19–22]. In cattle manure composts, alkyl-C first decreased, and then increased to a stable level in the curing stage, similar to the findings by Tang et al. [19], Gómez et al. [20] and Wang et al. [9]. In addition, alkyl-C in chicken manure composts generally increased as composting proceeded, coinciding with the results of Gómez et al. [20, 21] but inconsistent with those of Spaccini and Piccolo [22]. The increase of alkyl C may be attributed to the preservation of CH_2_ groups in long-chain paraffin structures [28], methylene groups in aliphatic rings and chains [29], and long-chain aliphatic bio-polyesters [30, 31].

#### O-alkyl C

O-alkyl C in cattle and chicken manure composts generally decreased as composting proceeded. The decrease of O-alkyl C was associated with the decomposition of easily degradable carbohydrates. The trend was in agreement with the findings in the study on cattle manure composts mixed with straw [19, 20], but contradictory to the finding from the composting of poultry manure composts mixed with straw [24]. The increase of O-alkyl C in their poultry manure composts may be due to enhanced microbial biomass and thus increased amino sugars. The O-alkyl C fluctuated in the theromphilic phase and slightly decreased until the end of composting in swine manure composts, inconsistent with other studies [9, 19, 20]. This was possibly attributed to the high content of carbohydrates in swine manure, and the relatively slower temperature increase in the thermophilic phase.

#### Aromatics and COO/NC = O

The increase of aromatic C and COO/NC = O C was found in the three manure composts during composting, which was in agreement with the findings of Gómez et al. [20] and Wang et al. [9], but contradictory to the results of Tang et al. [19]. The increase of COO could be due to the generation of carboxyl groups accompanied by oxidative degradation of organic matter [29]. For the three manure composts, the increase of the aromatic C during composting could be related to the humification [32, 33].

#### Aromatics C-O

In our study, aromatic C-O increased in cattle and swine manure composts but decreased in the chicken manure composts at the end of composting, which was not consistent with the results of Tang et al. [19], Gómez et al. [20] and Wang et al. [9]. The opposite change trend of aromatic C-O in chicken manure composts to that in cattle and swine manure composts is due to the degradation of polyphenols in chicken manure composts and the preservation of lignin in cattle and swine manure composts.

#### Alkyl C/O-alkyl C

**Fig 5:**
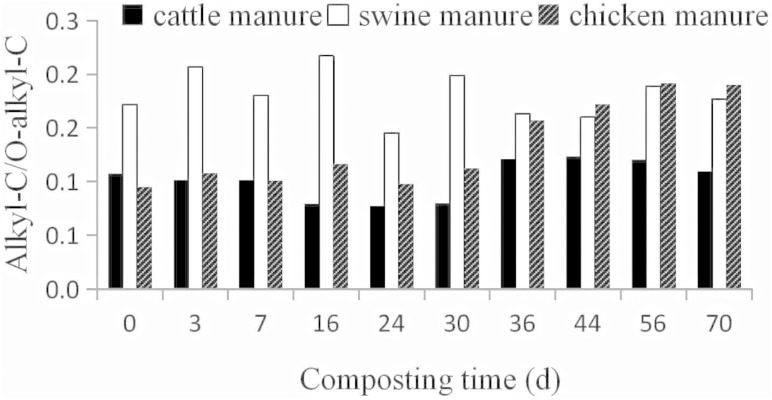
Alkyl C/ O-alkyl C ratios of manures and composts during composting. Alkyl C/ O-alkyl C = alkyl C [0-44ppm] / O-alkyl C [44–113 ppm] based on CP/TOSS spectra.

The alkyl C/O-alkyl C ratio was used as an indicator of the extent of decomposition, as suggested by Baldock et al. [18]. The change of alkyl C/O-alkyl C ratio (Fig 5) in the present study was not completely consistent with that of Tang et al. [19]. Tang et al. [19] reported the increase trends of alkyl C/O-alkyl C ratio of cattle manure mixed with rice straw during composting. We observed an increase trend in cattle and chicken manure composts but a fluctuant change trend was found in swine manure composts.

#### Aromaticity

**Fig 6:**
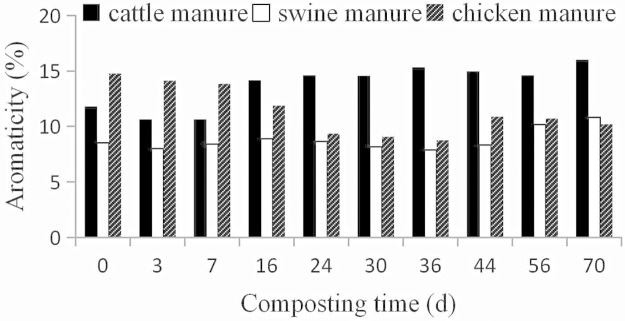
Aromaticity of manures and composts during composting. Aromaticity = aromatic C [113–162 ppm] / total C [0–220 ppm] based on CP/TOSS spectra.

Another index usually linked to humification is aromaticity [34, 35]. A useful parameter related to aromaticity is the nonprotonated aromatic carbon fraction. The nonprotonated aromatic carbon fraction was calculated using the CP/TOSS spectrum according to aromaticity = nonprotonated aromatic C [113–162 ppm] / total C [0–220 ppm]. The changes of aromaticity in three manure composts were also not completely consistent with those of Gómez et al. [20] or Wang et al. [9]. In the study of Gómez et al. [20], aromaticity first increased, and then decreased in composting of cattle and poultry manure mixed with straw. However, in the present study, we observed an increase trend of aromaticity in cattle and swine manure composts. But the aromaticity in chicken manure composts first decreased, and then increased, in agreement with the findings of Wang et al. [9]. The increase of aromaticity as composting proceeded was attributed to the preservation of lignin in cattle and swine manure composts, and the degradation of easily decomposed compounds such as carbohydrates and amino acids. The quick decrease of aromaticity in chicken manure composts (Fig 6) in the thermophilic phase was ascribed to the fast degradation of polyphenols.

### Maturity indices

A number of criteria and parameters have been proposed for assessing compost maturity, including physical characteristics such as color and temperature, chemical parameters such as pH, C/N, NH_4_^+^-N and NH_4_^+^-N/NO_3_^-^-N ratio, GI as well as structural information provided by ^13^C NMR. Here we propose that ideally a compost is mature if its chemical structure becomes relatively stable. Based on the NMR results (Figs 3 and 4), the chemical structure of the cattle manure compost at day 36 was quite similar to that at day 70 whereas the chemical structure of the chicken manure compost at day 56 was quite similar to that at day 70. However, obvious changes in chemical structure were still observed for swine manure composts from day 56 to 70. Therefore, we conclude that cattle and chicken composts are mature at day 36 and 56 but swine manure has not been mature up to day 70.

Our results from routine physicochemical analyses and NMR as well as GI can be compared with the threshold values of some maturity indices proposed in the literature (Table 2). It has been claimed that composts are of a good degree of stability when temperature reaches the ambient level during composting [18]. The ideal C/N ratios of starting materials for composting are in the range 25–35, and composts are regarded as being mature if their C/N ratios reach 20 or 12 [10, 36]. As mentioned above, composts were mature when their NH_4_^+^-N concentrations were lower than 400 mg/kg [16] and their NH_4_^+^-N/NO_3_^-^-N ratios lower than 0.16 [10]. Composts with GI values greater than 50% were regarded as being absent of phytotoxins [30]. The temperatures of all the three manure composts reached the ambient temperature at day 70. Since the initial C/N ratios of our three manures were lower than 25, the suggested C/N threshold ratio for maturity cannot be used to evaluate the degree of maturity in the present study. The concentration of NH_4_^+^-N below 400 mg/kg at day 44 and the NH_4_^+^-N/NO_3_^-^-N ratio below 0.16 at day 70 were found only in the cattle manure composts and GI values above 50% were obtained in all three manure composts at day 3. Therefore, the days required to reach the threshold values based on these maturity indices are different. The maturity based on physicochemical characteristics generally lagged behind of that assessed using NMR chemical structure information whereas the maturity evaluated using GI was ahead of that from NMR. The inconsistency implies that further investigations will be needed to find out the relationship between maturity indices based on physicochemical characteristics and those from chemical structures.

**Table 2:** Days required for three manures to reach the threshold values of some maturity indices proposed in the literature as well as threshold values and references.

Index	^13^C NMR	T (°C)	C/N	NH_4_^+^-N (mg kg^-1^)	Index	^13^C NMR
Swine manure composts	Beyond 70 days	70	0^a^ 0^b^	Beyond 70 days	Swine manure composts	Beyond 70 days
Cattle manure composts	36	70	0 ^a^ 0^b^	44	Cattle manure composts	36
Chicken manure composts	56	70	0 ^a^ 0^b^	Beyond 70 days	Chicken manure composts	56
Threshold value	N/A	Ambient temperature	<20 ^a^ <12^b^	<400	Threshold value	N/A
Reference	N/A	Satisha and Devaranjan [37]	Mathur et al. [36]^a^ Bernal et al. [10]^b^	Zucconi and Bertoldi [15]	Reference	N/A

N/A means not available.

^a^ from the reference of Mathur et al. (1993);

^b^ from the reference of Bernal et al. (1998).

## Conclusions

The pH change pattern of swine and chicken manure composts was not consistent with that of cattle manure composts and those in the literature. In addition the changing trend of pH significantly correlated with that of NH_4_^+^-N concentration. Significant N loss by ammonia volatilization in three manure composts occurred during composting, and therefore use of bulking agents are recommended for composting, if possible. The GI values increased rapidly in the first 7 days, and all reached 100% in three manure composts at the end of the composting.

^13^C-NMR indicated that O-alkyl C was predominant in cattle and chicken manure composts, whereas alkyl C was the major carbon structure in swine manure composts. Mineralization of O-alkyl C dominated the mature stage, and the increase of aromatic C and COO/NC = O C was generally found in the three manure composts during composting. Moreover, aromatic C-O in cattle and swine manure composts increased but that in the chicken manure composts decreased at the end of composting. Aromaticity first decreased and then increased in chicken manure composts, but increased in cattle and swine manure composts. The special change trend of aromatic C-O and aromaticity in chicken manure composts was due to the easily degradable polyphenol component. The FT-IR spectra did not show significant changes in chemical structures as composting proceeded for all three manures (S1 Fig).

The days required to reach the threshold values of maturity indices were different. Further investigations will be needed to find out the relationship between maturity indices based on physicochemical characteristics and those from chemical structures.

Solid-state ^13^C NMR combined with routine analyses have provided deep insights into the composting process of three manures. Three manures were of distinct properties, and their changes in physicochemical characteristics, chemical structures and maturity differed during composting. In addition, these changes were also different from those with addition of bulking agents. These strongly supports our hypothesis.

## Supporting information

S1 Fig FT-IR spectra of swine, cattle and chicken manure composts during composting. (TIF)

S1 Table Elemental compositions and C/N, C/H and C/O ratios of three manures and composts. (PDF)

S1 File Chemical structures and characteristics of animal manures and composts during composting. (PDF)

## Abbreviations

CP/TOSS: cross-polarization/total sideband suppression
CP/TOSS/DD: cross-polarization/total sideband suppression with dipolar dephasing
FT-IR: Fourier transform infrared
GI: Germination index
NMR: nuclear magnetic resonance
