# Supplementary material for: Chemical structures and characteristics of animal manures and composts during composting and assessment of maturity indices
Source: PLoS One. 2017 Jun 12;12(6):e0178110. doi: 10.1371/journal.pone.0178110 (PMC5467826; doi:10.1371/journal.pone.0178110)
Supplement: S1 Table — (PDF) [file pone.0178110.s002.pdf]

S1 Table Elemental compositions and C/N, C/H and C/O ratios of three manures and composts

	Time[d]	C%	N%	H%	O%	C/N	C/H	C/O
swine manure and composts	0	37.5	4.7	6.4	34.0	8.0	5.9	1.1
	3	36.8	4.8	6.4	35.3	7.7	5.7	1.0
	7	36.1	4.7	6.5	35.7	7.7	5.6	1.0
	16	32.7	4.7	6.2	34.6	7.0	5.2	1.0
	24	33.9	4.5	6.3	35.8	7.6	5.4	1.0
	30	30.8	4.2	6.1	34.4	7.4	5.1	0.9
	36	29.7	3.8	5.8	35.3	7.9	5.1	0.8
	44	29.4	3.9	5.7	33.0	7.6	5.2	0.9
	56	28.6	4.3	5.4	31.3	6.7	5.4	0.9
	70	28.6	4.4	5.3	32.8	6.5	5.4	0.9
cattle manure and composts	0	30.8	2.9	3.8	32.1	10.7	8.2	1.0
	3	30.8	2.7	5.0	34.8	11.3	6.2	0.9
	7	28.6	2.5	4.6	34.6	11.6	6.2	0.8
	16	26.4	2.5	4.3	34.0	10.5	6.1	0.8
	24	26.4	2.8	4.3	32.6	9.6	6.2	0.8
	30	29.4	2.7	4.6	34.8	10.9	6.4	0.8
	36	24.6	2.8	4.0	31.5	8.9	6.2	0.8
	44	25.2	2.9	3.8	27.7	8.7	6.6	0.9
	56	24.5	2.7	3.8	27.6	9.1	6.5	0.9
	70	24.4	2.7	3.8	26.8	8.9	6.4	0.9
chicken manure and composts	0	27.0	5.3	4.2	37.6	5.1	6.5	0.7
	3	26.5	5.0	4.1	38.5	5.3	6.4	0.7
	7	25.8	4.7	4.1	37.9	5.6	6.4	0.7
	16	23.3	3.7	3.7	37.2	6.3	6.2	0.6
	24	22.4	3.0	3.5	37.2	7.4	6.4	0.6
	30	20.2	2.3	3.2	35.5	8.7	6.4	0.6
	36	19.4	2.5	3.2	31.6	7.8	6.1	0.6
	44	19.5	2.6	3.2	29.6	7.5	6.2	0.7
	56	18.5	2.5	2.9	30.8	7.4	6.5	0.6
	70	19.5	2.9	3.2	30.3	6.8	6.0	0.6
