# Supplementary material for: Chemical structures and characteristics of animal manures and composts during composting and assessment of maturity indices
Source: PLoS One. 2017 Jun 12;12(6):e0178110. doi: 10.1371/journal.pone.0178110 (PMC5467826; doi:10.1371/journal.pone.0178110)
Supplement: S1 File — (PDF) [file pone.0178110.s003.pdf]

Supporting information

Chemical structures and characteristics of animal manures and composts during composting

Jieying Huang[1,2], Zixuan Yu[1], Hongjian Gao[1,2], Xiaoming Yan[3], Jiang Chang[1], Chengming Wang[3], Jingwei Hu[1] and Ligan Zhang[1,2]*

[1] School of Resources and Environment, Anhui Agricultural University, Hefei, Anhui，People's Republic of China

[2] Anhui Province Key Lab of Farmland Ecological Conservation and Pollution Prevention, Hefei, Anhui，People's Republic of China

[3] Institute of Agro-Products Processing Science and Technology, Anhui Academy of Agricultural Sciences, Hefei, Anhui，People's Republic of China

[4] Hefei National Laboratory for Physical Sciences at Microscale, University of Science and Technology of China, Hefei, Anhui，People's Republic of China

***Corresponding author**

E-mail: zhligan@ahau.edu.cn

Abbreviations Used

Germination index (GI); Fourier transform infrared (FT-IR); nuclear magnetic resonance (NMR); cross-polarization/total sideband suppression (CP/TOSS); cross-polarization/total sideband suppression with dipolar dephasing (CP/TOSS/DD).

Materials and methods

Sample preparation and samplings

For composting, the manures were put in a cool and ventilated place to allow them to dry naturally until their moisture contents reached about 60% (w/w). Then 12 kg each of three manures were composted separately in impervious plastic boxes (44 L volume, 53cm length x 38cm width x 22cm height), with three replicates for each manure. All the boxes were maintained in a temperature adjustable incubator for 70 days. The incubator was opened to ventilate for 15 minutes, and temperature recorded at the same time every day. The incubator temperature was adjusted to 1 ℃ below that of manure composts to reduce heat loss. During the first month, the manure composts were turned and mixed every day to provide aeration, and water was immediately added to maintain a moisture content of 60% (w/w). After one month, they were turned and mixed every other day to promote aerobic decomposition. The triplicate samples of each manure or compost were collected at day 0, 3, 7, 16, 24, 30, 36, 44, 56 and 70. A portion of the samples were air dried and ground through a 100-mesh sieve for NMR, FT-IR and elemental analysis, and the remaining portion stored in a refrigerator at - 20 ℃ for other analyses

Chemical analyses

The elemental compositions (%C, %N, %H, and %O) were determined by an Elementar Vario EL cube elemental analyzer through dry combustion at 1150 ℃ [1].

FT-IR spectroscopy

The FT-IR analyses were conducted on a Nicolet 8700 IR spectrometer. Each

sample was prepared by grinding 3 mg of the sample with 300 mg of oven-dried KBr in a vibrating puck mill and then compressing about 150 mg of the mixture into a translucent pellet under a hydraulic compressor. The pellet was immediately placed on a sample holder, and a spectrum ranging from 4,000 to 400 cm^{-1} was recorded under the conditions of 4 cm^{-1} wavenumber resolution and 32 scans. Pure KBr spectra were used as the background [2]. The major peaks (intensity and wavenumber) were found by using OMNIC software.

Statistical analysis

The FT-IR absorbance spectra were processed with OMNIC 8 spectroscopy software. The spectra were normalized by setting the highest value of each spectrum to 1 absorbance unit.

Results

Elemental compositions during composting

The changes in elemental compositions of the three manures and composts at different composting phases are shown in Table 1. Carbon was most abundant in all of the three manures. Swine manure had an obviously a higher carbon content (37.5%) than those of cattle manure (30.8%) and chicken manure (27.0%). Also chicken manure contained a higher nitrogen content (5.3%) than those of swine manure (4.7%) and cattle manure (2.9%). Swine manure had an obviously higher hydrogen content (6.4%) than those of cattle manure (3.8%) and chicken manure (4.2%). Moreover, chicken manure contained a higher oxygen content (37.6%) than those of swine manure (34.0%) and cattle manure (32.1%). Consequently, the C/N ratios of chicken

manure (5.1) and swine manure (8.0) were lower than that of cattle manure (10.7).

S1 Table 1 Elemental compositions and C/N, C/H and C/O ratios of three manures and composts

	Time[d]	C%	N%	H%	O%	C/N	C/H	C/O
swine manure and composts	0	37.5	4.7	6.4	34.0	8.0	5.9	1.1
	3	36.8	4.8	6.4	35.3	7.7	5.7	1.0
	7	36.1	4.7	6. 5	35.7	7.7	5.6	1.0
	16	32.7	4.7	6.2	34.6	7.0	5.2	1.0
	24	33.9	4.5	6.3	35.8	7.6	5.4	1.0
	30	30.8	4.2	6.1	34.4	7.4	5.1	0.9
	36	29.7	3.8	5.8	35.3	7. 9	5.1	0.8
	44	29.4	3.9	5.7	33.0	7.6	5.2	0.9
	56	28.6	4.3	5.4	31.3	6.7	5.4	0.9
	70	28.6	4.4	5.3	32.8	6.5	5.4	0.9
cattle manure and composts	0	30.8	2.9	3.8	32.1	10.7	8.2	1.0
	3	30.8	2.7	5.0	34.8	11.3	6.2	0.9
	7	28.6	2.5	4.6	34.6	11.6	6.2	0.8
	16	26.4	2.5	4.3	34.0	10.5	6.1	0. 8
	24	26.4	2.8	4.3	32.6	9.6	6.2	0.8
	30	29.4	2.7	4.6	34.8	10.9	6.4	0.8
	36	24.6	2.8	4.0	31.5	8.9	6.2	0. 8
	44	25.2	2.9	3.8	27.7	8. 7	6.6	0.9
	56	24.5	2.7	3.8	27.6	9.1	6.5	0. 9
	70	24.4	2.7	3.8	26.8	8.9	6.4	0.9
chicken manure and composts	0	27.0	5.3	4.2	37.6	5.1	6.5	0.7
	3	26.5	5.0	4.1	38.5	5.3	6.4	0.7
	7	25.8	4.7	4.1	37.9	5.6	6.4	0.7
	16	23.3	3.7	3.7	37.2	6.3	6.2	0.6
	24	22.4	3.0	3.5	37.2	7.4	6.4	0.6
	30	20.2	2.3	3.2	35.5	8.7	6.4	0.6
	36	19.4	2.5	3.2	31.6	7.8	6.1	0.6
	44	19.5	2.6	3.2	29.6	7.5	6.2	0.7
	56	18.5	2.5	2.9	30.8	7.4	6.5	0.6
	70	19.5	2.9	3.2	30.3	6.8	6.0	0.6

During the 70 days of composting, the carbon contents of the three manure composts gradually decreased. At the end of composting, the carbon content of swine manure composts decreased to 28.6%, still greater than those of the day-70 cattle manure compost (24.4%) and chicken manure compost (19.5%). The nitrogen content

of cattle manure composts slightly decreased in the first 16 days, increased to 2.8% and remained almost constant in the subsequent time. The nitrogen content of chicken manure composts rapidly decreased in the first 30 days and then slightly increased afterwards. However, the nitrogen content of swine manure composts was almost constant in the first 16 days, then decreased from day 24 to day 36, and increased in the remaining days. The hydrogen content of all the manure composts generally decreased. The oxygen content of cattle manure composts decreased in the first 24 days, increased to 34.8% at day 30, and then decreased until the end of composting. The oxygen content of chicken manure composts decreased to 29.6% in the first 44 days, and subsequently remained almost constant. But the oxygen content of swine manure composts was almost constant in the first 36 days, and decreased to 32.8% at the end of composting. The C/N ratio of chicken manure composts had an increasing trend whereas those of swine and cattle manure composts decreased during the composting. The C/H ratios generally decreased and the C/O ratios did not change much for all the three manure composts. All the differences described above were significant based on Duncan's multiple range tests at $P < 0.05$.

FT-IR analysis

S1 Fig FT-IR spectra of swine, cattle and chicken manure composts during composting

The FT-IR spectra of the cattle, swine and chicken manure composts at different stages (0, 7, 24, 44, and 70 days) exhibited similar spectral patterns (S1 Fig 1), except the differences in the intensity of certain bands. The main absorbance bands were those around 3300-3400 cm^{-1}, attributed to the intra-molecular and inter-molecular

hydrogen-bonded OH groups in phenol, carbohydrate and carboxylic acid compounds, as well as N-H stretching in amines and amides [6]. The two distinct small peaks at 2930 and 2854 cm^{-1} were ascribed to the stretching of aliphatic CH_3 and CH_2, respectively [7]. A pronounced peak at 1650 cm^{-1} was attributed to the C=O stretching of amide groups (amide I) and aromatic C=C vibrations, as well as to symmetric stretching of COO^- groups. A small peak at 1540cm^{-1} was assigned to vibrations in aromatic rings [8] and a distinct peak at 1430 cm^{-1} was due to the C-O asymmetric stretching, O-H deformation and C-O-H deformation of the carboxyl groups and symmetric stretching of the COO^- ions [6]. The small peak at 1230 cm^{-1} was attributed to C-O stretching and O-H deformation of carboxyls, phenols, and aromatic ethers; and a big band at 1030cm^{-1} is frequently assigned to C-H in-plane deformation vibration, C-O stretching of carbohydrates, polysaccharides or polysaccharides-like substances [9-11].

Spectral differences were observed among different manure composts. Compared with the spectra of the swine and cattle manure composts (S1 Fig(a) and (b)), the band around 3400 cm^{-1} became less intense and shifted toward a lower wavenumber at 3300 cm^{-1} in the spectra of the chicken manure composts (S1 Fig (c)). A small sharp peak at 2520 cm^{-1} was observed only in the spectra of chicken manure composts, which can be attributed to S-H stretch of aromatic or nonaromatic mercaptans and sulfides. Spectral differences were also observed in the 1540–1430 cm^{-1} region. The bands at 1540 cm^{-1} were distinct in the spectra of swine manure composts but smaller in the spectra of cattle and chicken manure composts. However, the bands at 1430

cm^{-1} were stronger in the spectra of chicken manure composts but weaker in the spectra of cattle and swine manure composts. The bands at 870, 765 and 612 cm^{-1} were distinct in the spectra of chicken manure composts but weaker in those of cattle and swine manure composts. These bands are preferentially assigned to vibrations in proton-substituted aromatic rings.

The FT-IR spectra did not differ noticeably among different composting stages. Only slight reduction of peaks in the aliphatic region at 2930 and 2854 cm^{-1} for cattle manure composts during the thermophilic phase was observed (S1 Fig (b)), possibly due to preferential biodegradation of aliphatic structures [12]. In addition a decrease of peak intensity in the aromatic region at 1650 cm^{-1} for chicken manure composts (S1 Fig (c)) and an increase of peak intensity in the polysaccharide region at 1030 cm^{-1} in swine (S1 Fig (a)) and chicken manure composts were observed as the composting proceeded.

Discussion

Evolution of elemental composition during composting

The elemental composition evolution of manures and composts during composting in the present study was similar to that of most other studies [13, 14], showing the decreased trends of C and N contents. However, Sánchez-Monedero et al. [15] and Huang et al. [16] observed the increase trend of N in humic acids as composting proceeded. They attributed the increase of N to incorporation of N in humic acids by condensation of lignin with proteins and formation of refractory, complex structures between nitrogen and the humic 'core'. The decreased trend of C/N

ratios for swine and cattle manure composts during our composting was also similar to that of other studies [13, 17-19]. Nevertheless, the change of C/N ratio for chicken manure composts in many studies was in disparity, with some showing the increase trend [13, 20] in agreement with our results but others indicating the decrease trend [17, 21]. Antil et al. [21] ascribed the decrease of C/N ratio to the decreased carbon content and increased N content in their study. The chicken manures and composts in the present study had greater N and pH but lower C/N ratio than swine and cattle manures and composts. The high pH in chicken manure composts contributed to faster losses of N relative to C via NH_3 volatilization [22] and thus as composting proceeded, the C/N ratio of chicken composts increased.

References

[1] Keeney DR, Nelson DW. Nitrogen: Inorganic Forms. Methods of Soil Analysis. Part 2-Chemical and Microbiological Properties, 2nd ed. ASA and SSSA, Madison, WI, USA, 1982; pp. 672-679.

[2] Haberhauer G, Gerzabek MH. Drift and transmission FT-IR spectroscopy of forest soils: an approach to determine decomposition processes of forest litter, Vib. Spectro. 1999; 19: 413-417.

[3] Zucconi F, Pera A, Forte M, Bertoldi MD. Evaluating toxicity of immature compost. Biocycle. 1981; 22: 54–57.

[4] Fujiwara T, Hara M, Murakami K. The Effect of Drying Method of Animal Waste Compost Samples on Evaluation of Plant Growth Inhibition, Journal of the Science of Soil and Manure Japan, 2003; 74: 607-614.

[5] Tang J, Nagamitsu M, Yutaka T, Arata K. Characterization of the maturing process of cattle manure compost. Process Biochem. 2006; 41: 380–389.

[6] Stevenson FJ. Humus chemistry: Genesis, composition, reactions. Wiley-Interscience, New York, 1982; pp. 443.

[7] Smidt E, Eckhardt KU, Lechner P, Schulten HR, Leinweber P. Characterization of different decomposition stages of biowaste using FT-IR spectroscopy and pyrolysis-field ionization mass spectrometry, Biodegrad. 2005; 16: 67-79.

[8] Hergert HL. Infrared spectra of lignin and related compounds. II. Conifer lignin and model compounds, J. Org. Chem. 1960; 25: 405-413.

[9] Giusquiani PL, Concezzi L, Busibelli M, Macchioni A. Fate of pig sludge liquid fraction in calcareous soil: agricultural and environmental implications, J. Environ. Qual. 1998; 27: 364–371.

[10] Olk DC, Brunetti G, Senesi N. Decrease in humification of organic matter with intensified lowland rice cropping: a wet chemical and spectroscopic investigation, Soil Sci. Soc. Am. J. 2000; 64:1337–1347.

[11] Mao JD, Ajakaiye A, Lan YQ, Olk DC, Ceballos M, Zhang TQ, et al. Chemical structures of manure from conventional and phytase transgenic pigs investigated by advanced solid-state NMR spectroscopy. J. Agric. Food Chem. 2008; 56: 2131-2138.

[12] Ghita A, José AA, José G, Juan C, Mohamed H. Chemical and spectroscopic analyses of organic matter transformations during composting of olive mill wastes, Int. biodeterior. biodegrad. 2004; 54: 39–44.

[13] Wang K, He C, You SJ, Liu W, Wang W, Zhang RJ, et al. Transformation of organic matters in animal wastes during composting. J. Hazard. Mater. 2015; 300: 745–753.

[14] Eneji AE, Yamamoto S, Honna T, Ishiguro A. Physico-chemical changes in livestock feces during composting, Commun. Soil Sci. Plant Anal. 2001; 32: 477–489.

[15] Sánchez-Monedero MA, Cegarra J, García D, Roig A. Chemical and structural evolution of humic acids during organic waste composting, Biodegradation. 2002; 13: 361–371.

[16] Huang GF, Wu QT, Wong JWC, Nagar BB. Transformation of organic matter during co-composting of pig manure with sawdust, Bioresour. Technol. 2006; 97:1834–1842.
[17] Hsu J, Lo S. Chemical and spectroscopic analysis of organic matter transformations during composting of pig manure. Environ. Pollut. 1999; 104: 189–196.
[18] Bustamante MA, Paredes C, Marhuenda-Egea FC, Pérez-Espinosa A, Bernal MP, Moral R. Co-composting of distillery wastes with animal manures: Carbon and nitrogen transformations in the evaluation of compost stability. Chemosphere. 2008; 72: 551–557.
[19] Brito LM, Mourão I, Coutinho J, Smith SR. Simple technologies for on-farm composting of cattle slurry solid fraction. Waste Manage. (Oxford). 2012; 32: 1332–1340.
[20] Tiquia SM, Tam NFY. Characterization and composting of poultry forced-aeration piles. Process Biochem. 2002; 37: 869–880.
[21] Antil RS, Raj D, Narwal RP, Singh JP. Evaluation of maturity and stability parameters of composts prepared from organic wastes and their response to wheat. Waste Biomass Valor. 2013; 4: 95-104.
[22] Morisaki N, Phae CG, Nakasaki K, Shoda M, Kubota H. Nitrogen transformation during thermophilic composting, J. Ferment. Bioeng. 1989; 67: 57–61.

Figure Captions

S1 Fig FT-IR spectra of swine, cattle and chicken manure composts during composting
